# Nucleic Acid Point-of-Care Testing to Improve Diagnostic Preparedness^[Author-notes ciac013-FM1]^

**DOI:** 10.1093/cid/ciac013

**Published:** 2022-01-07

**Authors:** Ilesh V Jani, Trevor F Peter

**Affiliations:** Instituto Nacional da Saúde, Marracuene, Mozambique; Clinton Health Access Initiative, Boston, Massachusetts, USA

**Keywords:** COVID-19, primary healthcare, diagnosis, point-of-care, nucleic acid test

## Abstract

Testing programs for severe acute respiratory syndrome coronavirus 2 have relied on high-throughput polymerase chain reaction laboratory tests and rapid antigen assays to meet diagnostic needs. Both technologies are essential; however, issues of cost, accessibility, manufacturing delays, and performance have limited their use in low-resource settings and contributed to the global inequity in coronavirus disease 2019 testing. Emerging low-cost, multidisease point-of-care nucleic acid tests may address these limitations and strengthen pandemic preparedness, especially within primary healthcare where most cases of disease first present. Widespread deployment of these novel technologies will also help close long-standing test access gaps for other diseases, including tuberculosis, human immunodeficiency virus, cervical cancer, viral hepatitis, and sexually transmitted infections. We propose a more optimized testing framework based on greater use of point-of-care nucleic acid tests together with rapid immunologic assays and high-throughput laboratory molecular tests to improve the diagnosis of priority endemic and epidemic diseases, as well as strengthen the overall delivery of primary healthcare services.

## Teaser

Rapid severe acute respiratory syndrome coronavirus 2 nucleic acid tests can improve COVID-19 control, overcoming limitations with laboratory polymerase chain reaction and rapid lateral flow tests. We propose a more optimized framework for use of these technologies to strengthen management of endemic and epidemic diseases.

Since the start of the coronavirus disease 2019 (COVID-19) pandemic, testing, contact tracing, and isolation have been the primary tools for severe acute respiratory syndrome coronavirus 2 (SARS-CoV-2) infection control. The early detection of emerging outbreaks or new variants is the first critical step in disease control. Quick testing of early suspected cases enables timely epidemic response actions to be taken, ultimately limiting the spread and impact of the disease. Significant investments have been made in the development and deployment of new diagnostics, and testing is expected to remain an essential intervention as COVID-19 vaccination programs scale up [1].

**Figure 1.**
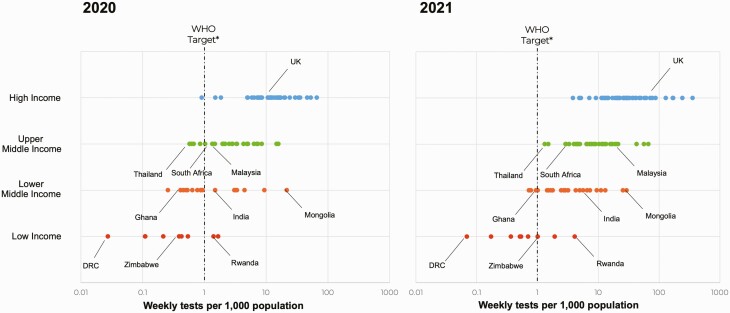
Inequity exists in access to testing: coronavirus disease 2019 (COVID-19) testing rates per 1000 vs gross domestic product per capita. Lower-income countries have conducted fewer COVID-19 tests to date than higher-income countries. Despite the deployment of rapid antigen assays since late 2020, the gap in testing rates continued to increase in 2021. Data source: Our World in Data [2].^∗^World Health Organization targets: 1 test per 1000 population per week [3]. Abbreviations: DRC, Democratic Republic of the Congo; UK, United Kingdom; WHO, World Health Organization.

However, testing rates have varied significantly across countries, with lower-income countries conducting fewer tests per capita than better-resourced countries, often below the benchmarks recommended by the World Health Organization (WHO) for epidemic control (Figure 1) [2, 3]. These differences have persisted since the start of the pandemic. During the first half of 2021, high-income countries conducted, on average, 52 tests per 1000 people per week with a positivity rate of 5.3%, while low- and middle-income countries (LMICs) conducted 13-fold fewer tests, with 4 tests per 1000 people per week and a positivity rate of 10.5%. While disease epidemiology, limitations in funding and test supplies, and other constraints have contributed to this difference [4, 5], inadequate technologies for delivering testing services at the primary healthcare level has been a major determining factor.

## WEAKNESSES OF COVID-19 DIAGNOSIS IN LOW-RESOURCE SETTINGS

An effective control of emerging epidemics requires capacity to detect and respond during the early stages of outbreaks. This capacity sits at the core of primary healthcare where >80% of people first seek medical care. In many LMICs, the majority of primary healthcare facilities are small health centers and clinics that have a limited menu of onsite tests [6]. During most of the first year of the COVID-19 pandemic, these facilities relied on referring samples to central laboratories for polymerase chain reaction (PCR) testing, and then started to use rapid antigen tests as these became available. While the availability of reference laboratory PCR and rapid antigen tests has been important, the lack of onsite diagnostic capacity at the primary healthcare level for many months hampered early opportunities for COVID-19 control.

Reference laboratories were the first to diagnose SARS-CoV-2 infections based on PCR as they had the necessary infrastructure, equipment, and personnel. The early sequencing of the virus made it possible for molecular probes to be designed and manufactured within weeks of the occurrence of the first identified SARS-CoV-2 cases [7]. For the first year of the pandemic, these laboratories and other high-volume facilities conducted the majority of SARS-CoV-2 tests and are still the backbone of many national testing programs. However, most PCR testing platforms are large, costly instruments that require sophisticated laboratories with stable electricity, climate control, and specialized software and skills to run, and are often located in larger urban areas. While these reference laboratories are necessary to handle complex surveillance activities and testing demand during high-transmission waves, inherent delays in turnaround time may undermine their effectiveness for disease control. Modeling studies suggest that results returned and acted on within 1–2 days of sample collection have the highest impact on limiting the spread of infection [8]. This is operationally difficult to achieve, especially where there is a need to ship samples from primary healthcare facilities in periurban and rural areas where large proportions of the population reside in low-income countries. Test turnaround times of 3–5 days have been reported in low-resource settings, whereas in higher-income settings PCR test turnaround times have often been greater than 2 days [9–13]. While an essential tool, central laboratory PCR has not adequately served the needs of early epidemic detection and control in many geographical locations.

Access to onsite rapid SARS-CoV-2 testing was limited during the first year of the pandemic. Few health facilities had point-of-care PCR test capacity, and supply shortages restricted the use of rapid PCR assays for COVID-19 in LMICs [4, 5]. While SARS-CoV-2 lateral flow rapid antigen tests are now widely used, these tests were slower to develop and validate than nucleic acid assays. The first WHO emergency-use listed rapid antigen tests became available in September 2020 [14, 15]. Uptake of these tests in LMICs has been gradual because of local regulatory approvals and testing policy updates, funding gaps, and the need to train healthcare workers and implement these tests across thousands of health facilities. While efforts to deploy rapid antigen tests at community level are underway [16], concerns around their analytical performance have restricted their use to symptomatic and high-risk persons such as healthcare workers and close contacts. Rapid antigen tests were not recommended by the WHO for point-of-entry and asymptomatic person screening due to concerns around lower sensitivity and specificity [17, 18]. As a result of these factors, low-resource settings have to date lacked low-cost, high-performing, and widely accessible SARS-CoV-2 tests that are approved for the majority of use cases. Importantly, inexpensive point-of-care nucleic acid tests have not been available.

This technological gap, particularly in primary healthcare, is a persistent flaw in the diagnostic preparedness of LMICs and is likely to hinder rapid control of COVID-19 as well as of other epidemic infections. As COVID-19 control evolves and health systems consider long-term plans for improving diagnostic capacity, point-of-care nucleic acid assays need to become routinely available within primary healthcare to bridge the gap between laboratory-based PCR and rapid lateral flow testing.

## POINT-OF-CARE NUCLEIC ACID TESTING FOR ENDEMIC AND PANDEMIC DISEASES

Point-of-care nucleic acid technologies are relatively new and have not been widely deployed to date. Their use has been recommended in order to improve test access and clinical outcomes in ways that complement conventional laboratory testing. Prior investments in tuberculosis (TB) and human immunodeficiency virus (HIV) control have promoted the use of these technologies, but coverage remains low. For example, <20% of public health facilities surveyed across 7 sub-Saharan African countries have point-of-care nucleic acid diagnostic capacity [19]. Cost, operational challenges, and limited technological options have so far restricted the expansion of this type of rapid molecular testing [20–24].

Novel point-of-care nucleic acid tests are under development and offer the opportunity to lower the barriers to wider use. These include device-based and disposable technologies, both of which are likely to be important and provide complementary benefits in different settings. A number of initiatives are investing in these technologies, including the WHO-led Access to COVID-19 Tools Accelerator (ACT-A), the FIND-led global alliance for diagnostics, the US National Institutes of Health Rapid Acceleration of Diagnostics (RADx) program, and the Bill & Melinda Gates Foundation [25, 26]. Although not all of these technologies are likely to be successfully adopted, the pipeline of products is promising.

Placing nucleic acid point-of-care technologies at major primary healthcare facilities and district hospitals offers the opportunity to establish a more widespread infrastructure of low-cost, multidisease point-of-care nucleic acid testing capacity closer to patients. Since the design of reagents for these molecular assays is based on genetic sequences of the pathogen, new point-of-care tests for detection of emerging infections or variants could be rapidly developed, preferably at the same time as new PCR tests for central laboratories within weeks of first detection [27].

For rapid nucleic acid technologies to be used widely, they need to be low-cost, easy to use and maintain, and functional across a wide set of environmental conditions. Ideally, each technology should also be capable of testing for a number of endemic infections such as TB, HIV, and human papillomavirus (HPV). Building capacity for rapid nucleic acid testing at primary healthcare will not be easy but has begun [28]. Over the past 10 years, these technologies have been deployed at thousands of health facilities, but often only at <20% of the primary healthcare footprint in lower-income countries [19, 28].

Improving nucleic acid testing capacity at the primary healthcare level can help address diagnostic gaps for endemic diseases. Inadequate access to accurate diagnosis continues to limit the health gains of investments in the control of endemic infections such as HIV, HPV, and viral hepatitis. For example, the WHO recommends the use of point-of-care HIV nucleic acid assays for pregnant and breastfeeding women and for the diagnosis of HIV infection in infants [29]. Greater use of point-of-care viral load tests to manage HIV infection may also help reduce the emergence of SARS-CoV-2 variants within immunocompromised populations. However, these tests and their associated health benefits are not accessible for the majority of patients in LMICs. Only two-thirds of HIV-exposed infants are tested within 2 months of life and, of these, it is estimated that <10% receive a point-of-care test [30, 31]. The United Nations resolution to eliminate cervical cancer is dependent on high coverage of screening and treatment programs that could benefit from more widespread access to low-cost HPV nucleic acid tests, ideally conducted at the point of care to enable same-day screening and treatment [32, 33]. In LMICs, where >80% of cervical cancer cases occur, current screening coverage is on average 20%, well below the 70% by 2030 target set by WHO and the nearly 80% current coverage in high-income countries [34, 35]. TB control would also benefit from more widely available, high-accuracy and low-cost onsite molecular assays to increase testing coverage and improve on the low reliability of the nonmolecular diagnostic methods used for the majority of TB patients. Currently, only a third of new TB cases are diagnosed using point-of-care nucleic acid tests and new tests will provide an opportunity to increase stagnated treatment rates and reduce incidence [28]. Establishing a common point-of-care infrastructure that can be used for different diseases will be important. Multidisease testing with existing rapid nucleic acid technologies has been shown to be feasible and necessary to accommodate increased test demand related to COVID-19 [36, 37].

## A FRAMEWORK FOR DIAGNOSTIC PREPAREDNESS

**Figure 2.**
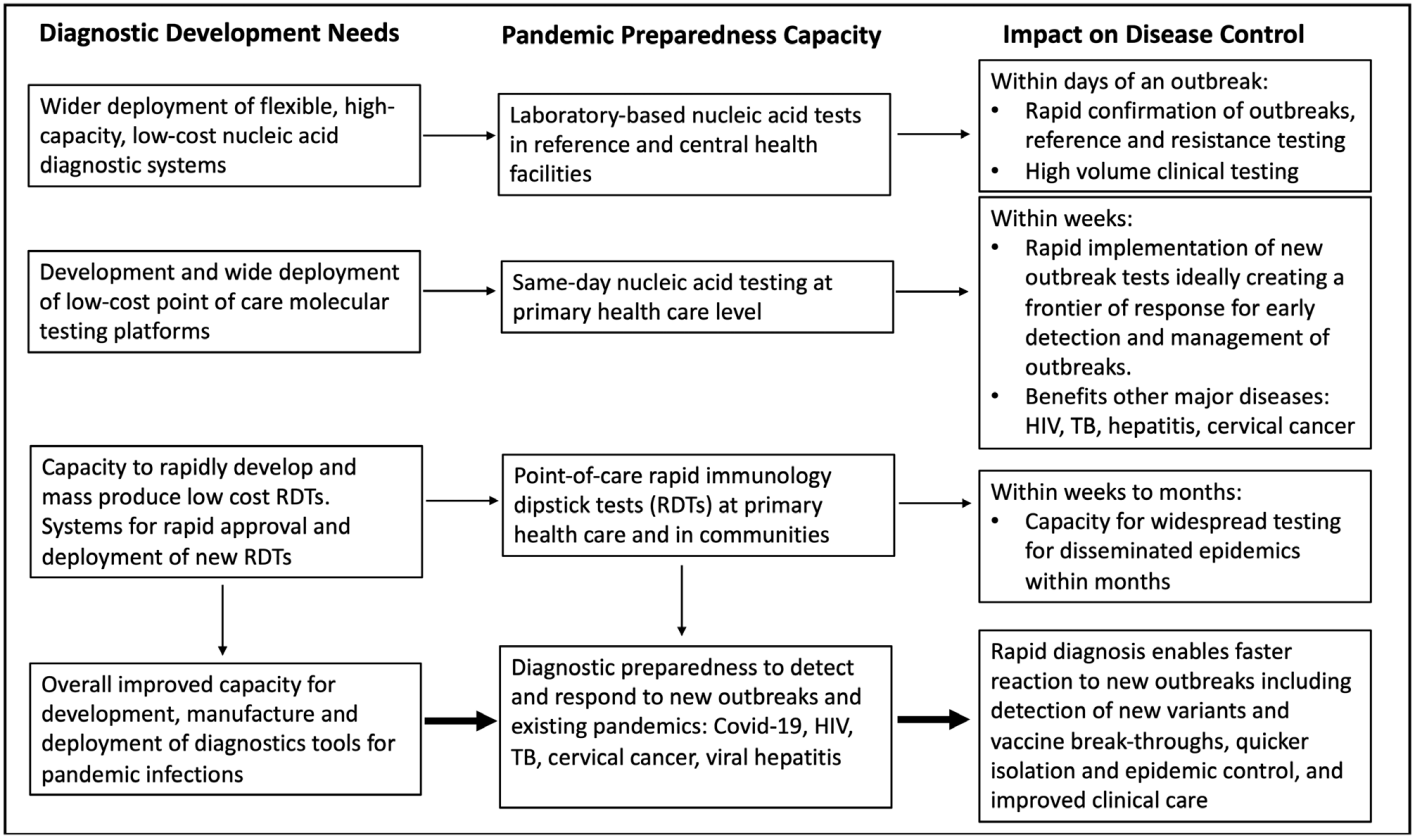
Framework for diagnostic preparedness in pandemic disease control. Preparedness for pandemic diseases requires the rapid availability of diagnostics in 3 distinct but interconnected levels. Diagnosis in reference laboratories and central health facilities should be performed using high-throughput technology. Primary healthcare facilities, which serve as the main interface between health services and communities, have the most significant diagnostic gap and should widely implement point-of-care nucleic acid assays to enable same-day testing of epidemic and endemic diseases. Mass screening at community and primary healthcare levels should be conducted using rapid immunological assays that can be developed and deployed rapidly. Abbreviations: COVID-19, coronavirus disease 2019; HIV, human immunodeficiency virus; RDT, rapid diagnostic test; TB, tuberculosis.

Diagnostic preparedness refers to the capacity of the health system to address the diagnostic needs of existing and emerging threats. We propose below a framework to strengthen preparedness across the health system using a balanced combination of technologies, with particular emphasis on primary healthcare (Figure 2, Table 1).

**Table 1.** Comparison of Diagnostic Technologies for Endemic and Epidemic Disease Detection, and Priorities to Improve their Utility for Pandemic Preparedness

Technology	Advantages	Drawbacks	Priorities for Improvement
Central laboratory NATs	High sensitivity and specificity, high throughput, multiplex across diseases	Limited access outside of centralized locations with laboratory infrastructure, slow test turnaround time	• Expand use of lower-cost, high-volume PCR systems with flexible test menus that enable rapid development and use of new assays • Rapid sample transport and electronic results delivery systems
Point-of-care NATs	High sensitivity and specificity, detection near patient, fast turnaround time	Lower throughput, few technologies available with limited deployment to date, potentially high cost	• Deploy routine multidisease tests across >80% of primary healthcare facilities • Prioritize low-cost and easy-to-use platforms • Use both simple device-based and instrument-free technologies
Rapid immunologic tests	Low cost and higher flexibility to deploy in most settings	Lower sensitivity and higher risk of test errors due to manual operation	• Build systems for rapid development, validation, and deployment of novel rapid tests • Standardize test formats to reduce training requirements • Expand use of data systems to transmit test results for disease tracking

Abbreviations: NAT, nucleic acid test; PCR, polymerase chain reaction.

First, health systems should have a reliable network of reference laboratories with flexible nucleic acid platforms capable of high-volume testing as well as early diagnosis of fresh outbreaks of SARS-CoV-2 and its variants, as well as new emerging threats. These laboratories should be linked to sequencing facilities that are part of global surveillance systems tracking the emergence of new infections and variants. These facilities need rapid and widely accessible sample referral and results delivery systems and should also conduct testing for other epidemic and endemic diseases. Reference laboratories will also continue to play important roles in promoting point-of-care testing at primary healthcare through test setup, training, and ongoing management of supportive systems such as data management, supply chain, and quality assurance.

However, these laboratories suffer from high costs, difficult supply and limited flexibility in test menu, and poor systems for sample referral and results delivery [38, 39]. Technologies such as open PCR may help laboratories lower costs, expand test menus, and make nucleic acid testing more routinely available in less-resourced settings with high disease burdens. In LMICs, open PCR platforms that were previously dedicated to research or surveillance for influenza and other diseases were used to rapidly establish the capacity for SARS-CoV-2 detection from the outset of the pandemic. These systems have since become the mostly widely used systems for nucleic acid–based SARS-CoV-2 testing in LMICs due to lower cost, a wider range of suppliers, and flexible instrumentation. Open PCR may enable high-volume, low-cost molecular testing for multiple diseases, provided operational challenges with less automated testing systems are overcome [40].

Second, the global community should strengthen the capacity for the rapid manufacture and deployment of low-cost, high-quality rapid lateral flow assays for large-scale testing at primary healthcare and in community settings. In order to be effective for epidemic control, fast-track systems for test development, validation, regulatory approval, large-scale manufacturing, and training are needed, deployable ideally within the first few weeks to months of an outbreak. While the emergency-use approval systems deployed early in the pandemic by the WHO provided valuable guidance for accelerated national regulatory approvals at a time when limited data were available, much shorter data generation and regulatory approval timelines at the international and local levels are needed for novel diagnostics to enable effective disease control. To enable faster deployment, a new generation of rapid tests is needed based on a more standardized format to reduce the need for extensive retraining of healthcare workers, and these need to be made available at low cost at the outset to allow mass testing early in the course of new epidemics. In addition, to track new cases identified by rapid testing and ensure test quality, improved data systems that automatically capture and transmit test results to disease surveillance and other health management systems are needed, building on early experiences with COVID-19, malaria, and HIV [41–43]. Besides COVID-19, the control of other epidemic-prone diseases such as cholera, yellow fever, dengue, measles, and influenza would benefit from this capacity. Moreover, the diagnosis of endemic diseases such as HIV, malaria, and TB that relies extensively on existing rapid immunological tests, and for which novel lateral flow technologies such as CD4 and TB-lipoarabinomannan assays are being explored, would also profit from novel data systems.

Third, healthcare systems need to expand the use of multidisease point-of-care nucleic acid testing to the majority of primary healthcare facilities in order to support early outbreak detection and cater to testing needs of endemic diseases. The limited availability of technologies suitable for deployment in low-resource settings, combined with the complexities of implementing such platforms, has made this the most significant gap. The emergence of a new generation of rapid nucleic acid technologies should facilitate improvements in diagnostic capacity for both endemic and emerging epidemic diseases at primary healthcare. While this will enable a further shift toward universal, routine nucleic acid testing, it will need to be accompanied by efforts to reduce operational, cost, and test performance barriers that have previously hampered the availability of point-of-care molecular diagnostics [20–24]. Regulatory, operational, and policy barriers to new diagnostic tests are persistent challenges that limit the pace and effectiveness of outbreak responses, often due to inadequate resources and prioritization. Consistent investment and innovation in these processes are needed to ensure efficiency and readiness even in the absence of active outbreaks. In addition, investments made in the development of new technologies will need to be complemented with support for early-stage commercialization and market entry, as this stage is the most perilous for novel diagnostic products in low-resource settings. This includes support for final clinical trials and regulatory approvals, commitments to secure supply for LMICs, and where needed, guaranteed minimum procurement volumes to facilitate commercial viability during early stages of adoption and scale-up when demand is uncertain and unpredictable. Last, it will also be important that these new technologies are not restricted to disease control programs with the most funding, but are part of a common health infrastructure to support testing across multiple diseases.

## CONCLUSIONS

COVID-19 has brought pandemic preparedness and the need to strengthen health security into sharper focus. The use of rapid nucleic acid tests has been vastly inadequate to enable early detection of new outbreaks and for the control of endemic diseases. As new rapid nucleic acid technologies become available, it will be important to expand the use of such technologies in primary healthcare where diagnostic capacity has been most limited and where cases of COVID-19 and other new disease outbreaks present first. The global health community has a unique opportunity to simultaneously resolve the persistent inequities in access to diagnostics and invest in the preparedness necessary to deal with both existing and emerging diseases. Deprioritizing rapid nucleic acid tests in primary healthcare yet again will perpetuate weaknesses in the control of COVID-19 and in preparedness for new epidemics, as well as prolong our inability to overcome the burden of endemic diseases.

## Notes

***Acknowledgments.*** The authors acknowledge the help of Owen Demke, Julia Tuttle, Constance McDonough-Thayer, and Paolo Maggiore in providing data and analyses for this article.

***Financial support.*** This work was supported by Unitaid and the Bill & Melinda Gates Foundation (funding opportunity number OPP1214435).
